# Determination of heavy metals in the soils of tea plantations and in fresh and processed tea leaves: an evaluation of six digestion methods

**DOI:** 10.1186/s13065-016-0154-3

**Published:** 2016-02-18

**Authors:** Md. Harunur Rashid, Zeenath Fardous, M. Alamgir Zaman Chowdhury, Md. Khorshed Alam, Md. Latiful Bari, Mohammed Moniruzzaman, Siew Hua Gan

**Affiliations:** Agrochemical and Environmental Research Division, Institute of Food and Radiation Biology, Bangladesh Atomic Energy Research Establishment, Savar, Dhaka, 1349 Bangladesh; Food Analysis and Research Laboratory, Center for Advanced Research in Sciences, University of Dhaka, Dhaka, Bangladesh; Department of Pharmacology, School of Medical Sciences, Universiti Sains Malaysia, Kubang Kerian, 16150 Kota Bharu, Kelantan Malaysia; Human Genome Centre, School of Medical Sciences, Universiti Sains Malaysia, Kubang Kerian, 16150 Kota Bharu, Kelantan Malaysia

**Keywords:** Fresh tea, Black tea, Heavy metals, Nitric acid, Hydrogen peroxide, Perchloric acid, Dry ashing, GF-AAS

## Abstract

**Background:**

The aim of this study was to determine the levels of cadmium (Cd), chromium (Cr), lead (Pb), arsenic (As) and selenium (Se) in (1) fresh tea leaves, (2) processed (black) tea leaves and (3) soils from tea plantations originating from Bangladesh.

**Methods:**

Graphite furnace atomic absorption spectrometry (GF-AAS) was used to evaluate six digestion methods, (1) nitric acid, (2) nitric acid overnight, (3) nitric acid–hydrogen peroxide, (4) nitric–perchloric acid, (5) sulfuric acid, and (6) dry ashing, to determine the most suitable digestion method for the determination of heavy metals in the samples.

**Results:**

The concentration ranges of Cd, Pb, As and Se in fresh tea leaves were from 0.03–0.13, 0.19–2.06 and 0.47–1.31 µg/g, respectively while processed tea contained heavy metals at different concentrations: Cd (0.04–0.16 µg/g), Cr (0.45–10.73 µg/g), Pb (0.07–1.03 µg/g), As (0.89–1.90 µg/g) and Se (0.21–10.79 µg/g). Moreover, the soil samples of tea plantations also showed a wide range of concentrations: Cd (0.11–0.45 µg/g), Pb (2.80–66.54 µg/g), As (0.78–4.49 µg/g), and Se content (0.03–0.99 µg/g). Method no. 2 provided sufficient time to digest the tea matrix and was the most efficient method for recovering Cd, Cr, Pb, As and Se. Methods 1 and 3 were also acceptable and can be relatively inexpensive, easy and fast. The heavy metal transfer factors in the investigated soil/tea samples decreased as follows: Cd > As > Se > Pb.

**Conclusion:**

Overall, the present study gives current insights into the heavy metal levels both in soils and teas commonly consumed in Bangladesh.

## Background

Tea (*Camellia sinensis L.*) is one of the most popular nonalcoholic beverages, consumed by over two-thirds of the world’s population for its medicinal, refreshment and mild stimulant effects [1]. Tea leaves contain polyphenols such as epigallocatechin 3‐gallate, which has many medicinal properties, including antioxidant [2], cholesterol-lowering [3], hepatoprotective [4] and anticancer activities [5]. Moreover, its detoxifying properties are essential in the elimination of alcohol and toxins [5]. However, considering that an estimated 18 billion cups of tea are consumed daily worldwide [6], its economic and social importance is unprecedented. In fact, tea has been reported to be valuable in the treatment and prevention of many diseases [6].

Ideally, tea should be free from contaminants such as heavy metals, which are toxic and harmful to the human body because of their non-biodegradable nature, long biological half-lives and persistent accumulation in different body parts [7]. Tea is consumed in all of Bangladesh throughout the year, and Bangladesh is one of the leading tea producing and exporting countries in the world [8]. In 2006, Bangladesh exported approximately 5 million kg of tea leaves, and this figure continues to increase even while the total local tea consumption in the country is reported to be 39 million kg [8].

Tea processing and packaging in Bangladesh is dependent on the type of tea, with a wide variety available in the country that is produced by different processing steps. However, the common steps involve the (1) hand plucking of tea leaves by the local farmers, (2) the weighing of tea leaves and (3) transportation to factories. Freshly plucked tea leaves are fragile, and as the first step in processing, the leaves are laid out to dry for several hours to allow them to “wither” as their moisture content decreases. The leaves are then rolled and oxidized, which alters their flavor and gives the processed tea its final appearance and color. The above steps are also known as Crush-Tear-Curl (CTC). The next step involves firing (final drying process), a process that is initiated once the tea leaves have dried. This is followed by visually sorting into various batches of similar sizes and color before being packaged and commercialized both nationally and internationally. For black tea, the leaves are rolled immediately after withering to quickly initiate the oxidation or fermentation processes. The leaves are then completely oxidized before they are dried, which is how they acquire their dark color and rich flavor.

Tea safety has piqued great interest because contaminants threaten the life and health of humans, animals and the environment, leading to economic losses [2]. The genetic and epigenetic effects of dietary heavy metals such as cadmium (Cd), chromium (Cr), lead (Pb), arsenic (As) and selenium (Se) in the human body are associated with an increased risk of different cancers [9]. Prolonged consumption of heavy metals from food can lead to their accumulation in the kidney and liver, causing disruption of numerous biochemical processes and potentially causing cardiovascular, nervous, kidney and bone diseases [10].

Elemental analysis of a tea sample requires destruction of the organic fraction of the sample, leaving the heavy metals either in solution or in a form that is readily dissolved. Unfortunately, because of a large number of analytes and a variety of sample types, there is no universal sample preparation technique that meets all of the diverse requirements. Among the strategies for sample preparation, dilution, acid digestion and extraction are the most commonly considered [11–20]. Microwave digestion, wet digestion and dry ashing are commonly utilized for the total decomposition of organic matter in samples [11, 21, 22]. Apart from these techniques, ultrasound-assisted solubilisation/extraction sample preparation procedures were reported to be used for green and black tea samples [23].

Dry ashing consists of the ignition of organic compounds by air at atmospheric pressure and at relatively elevated temperatures (450–550 °C) in a muffle furnace. The resulting ash residues are dissolved in an appropriate acid. Wet digestion is used to oxidize the organic portion of samples or to extract elements from inorganic matrices by means of concentrated acids or mixtures there of [24]. Compared to dry ashing, wet digestion may be performed with a wide variety of potential reagents. Although many types of acids, including hydrochloric acid (HCl), nitric acid (HNO_3_), sulfuric acid (H_2_SO_4_), perchloric acid (HClO_4_), and hydrogen peroxide (H_2_O_2_), are used to digest organic samples and soils [11, 25], it remains undetermined which type of acid/acid mixture is the most suitable.

In addition, little is known about the relative recovery of heavy metals from tea leaves, and there are no standard official methods in Bangladesh for the digestion of tea to determine heavy metals. Moreover, to our knowledge, there is limited data on the amount of heavy metals in fresh tea leaves, processed tea or soils from tea plantations in Bangladesh. Therefore, the aims of this study were (1) to determine the concentrations of common heavy metals such as Cd, Cr, Pb, As and Se in tea leaves and soils from tea plantations; (2) to report the degree of contamination and daily intake of toxic heavy metals via tea (3); to measure the interaction of heavy metal concentrations in fresh tea leaves, processed tea and soils from tea plantations by analyzing the transfer factor (TF); and (4) to evaluate six digestion methods using different acid combinations and recommend the most appropriate digestion method for determining the levels of five heavy metals in tea samples.

## Experimental

### Chemicals and reagents

Heavy metal reference standards for Cd, Cr, Pb, As, and Se were purchased from Kanto Chemical (Tokyo, Japan). Digestion chemicals including HCl, HNO_3_, H_2_SO_4_, HClO_4_, and H_2_O_2_ were of analytical grade and were purchased from Merck (Darmstadt, Germany).

### Description of study area

**Fig. 1:**
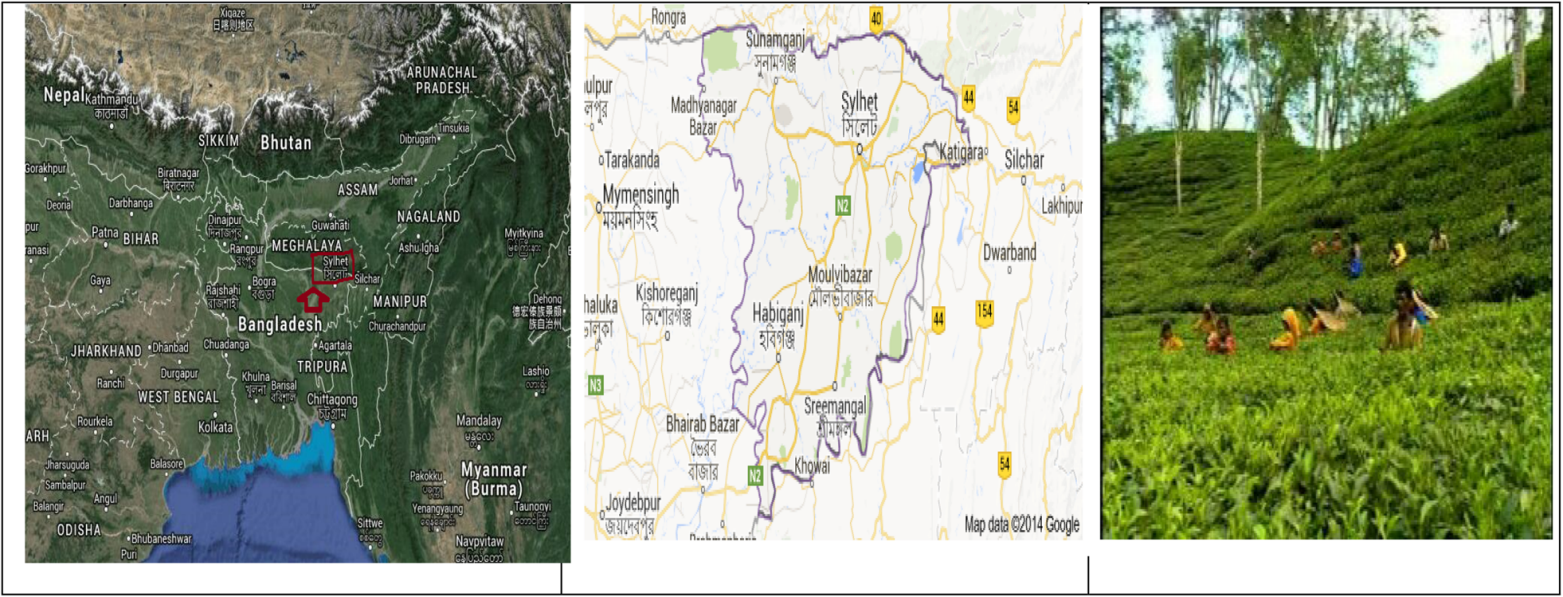
Sampling location of tea gardens and leaves

The samples were collected from two main tea growing areas (Moulvibazar and Sylhet) (Fig. 1). Moulvibazar is also known as the capital of tea production in Bangladesh, with miles and miles of tea gardens that look like green carpets. These areas have over 150 tea gardens, including three of the largest tea gardens in the world both in area and production.

### Collection and preservation of samples

**Fig. 2:**
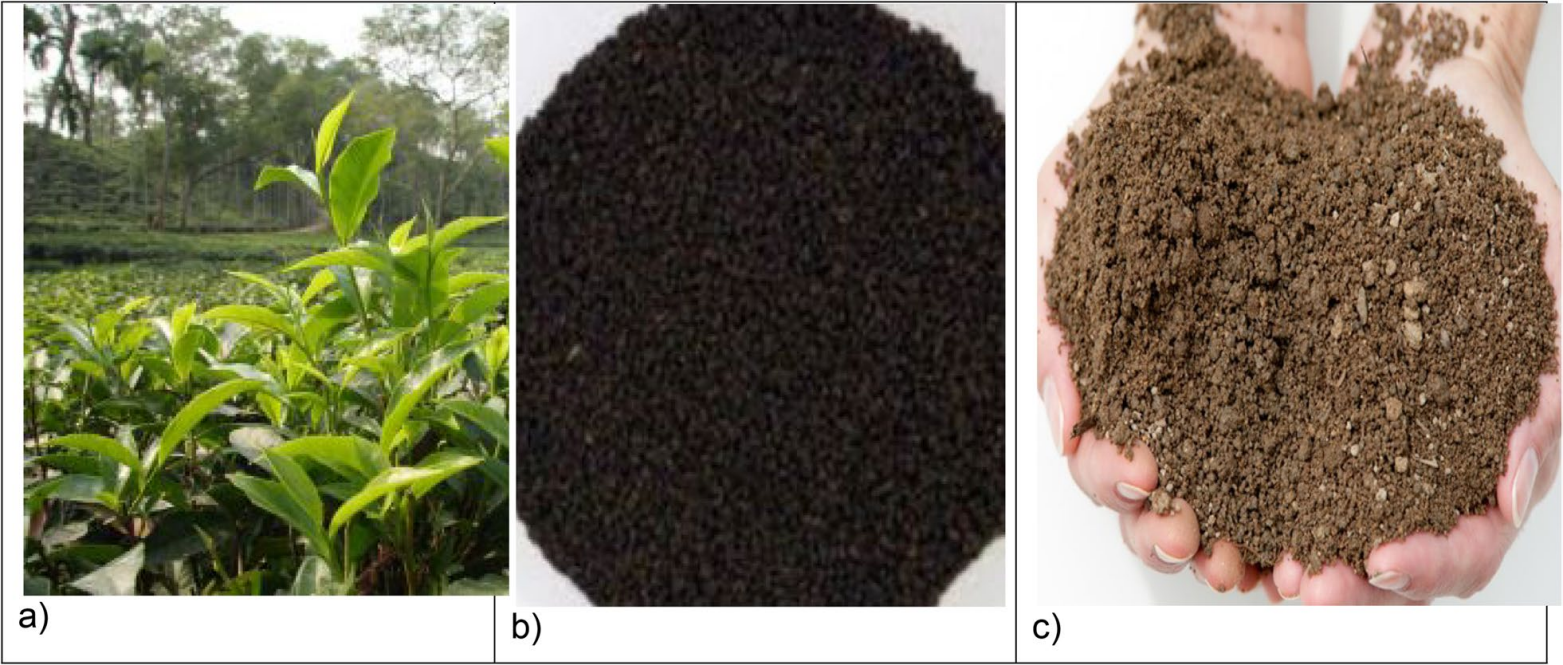
The investigated samples of (**a**) fresh tea leaves (**b**) processed/black tea and (**c**) soils from the tea plantations

Fresh tea leaves (n = 10) were randomly collected from five different tea gardens in the Sylhet district (n = 5), with the remaining from the Moulvibazar district (n = 5) (Fig. 2). Each collection consisted of 500 g of tea leaves and was authenticated by a botanist. For black tea, five processed tea samples were randomly purchased from the local market in Moulvibazar, with another five from the local market in Sylhet. The samples were supplied by the local tea gardens from the same areas. Purchased tea sample were processed by plucking, withering, rolling, oxidation and firing. First, the leaves were harvested by hand. After plucking, the leaves were laid out to wilt or wither for several hours to prepare for further processing. During withering, the leaves were gently fluffed, rotated and monitored to ensure that an even exposure to air. Then, the leaf was put through a rolling machine to mince, twist and break it into even smaller pieces. After rolling, the leaves were laid out to rest for several hours, allowing oxidation (the process in which oxygen in the air interacts with the exposed enzymes in the leaf, turning the sample to a reddish-brown color and changing the chemical composition) to occur. This step also has the greatest impact in the creation of the many wonderful and complex flavors in tea. The final step in the production process is to “fire” or heat the leaves quickly to dry them to below 3 % moisture content and to stop the oxidation process to ensure that the tea samples were kept well. During rolling and withering step of tea processing, tea may be considered to be contaminated.

Soils from tea plantations (n = 10) were randomly collected from locations similar to where the 10 fresh tea leaf samples were collected (from both Sylhet and Moulvibazar districts, Bangladesh). The soil samples (sandy clay loam) were collected (500 g each time) close (1–10 cm perimeter) to the tea plant by digging into the soil (1–5 cm depth). Some of the tea gardens were located near a highway (the closest was within 100 meters), and others were situated very far from the highway.

The collected samples were stored in clean, sterile polyethylene bags and were properly labeled. They were immediately sent to the laboratory of the Agrochemical and Environmental Research Division, Bangladesh Atomic Energy Commission, Dhaka, and were stored at −20 °C to reduce the risk of hydrolysis or oxidation prior to analysis.

### Digestion of samples

#### Digestion of tea samples

Before sample digestion, the tea leaves were freeze-dried at −50 °C at 100 Pa for 24 h. They were then crushed using a sterile mortar and pestle and sieved (particle size <100 µm) at room temperature. Finally, 1 g of tea leaves was used for digestion (refer to the six digestion methods described below).

#### Digestion of soil samples

Soil samples were oven dried at 60 °C for 24 h before being ground into a fine powder using a sterile mortar and pestle. The samples (2.5 g) were transferred into a crucible before being mixed with 10 mL of aqua regia, which consisted of HCl:HNO_3_ (3:1). The mixture was the digested on a hot plate at 95 °C for 1 h and was allowed to cool to room temperature. The sample was then diluted to 50 mL using deionized distilled water and was left to settle overnight [26]. The supernatant was filtered through Whatman No. 42 filter paper and (<0.45 µm) Millipore filter paper, (Merck Millipore, Darmstadt, Germany) prior to analysis by graphite furnace atomic absorption spectrometry (GF-AAS).

#### Method 1 (HNO_3_ digestion)

Based on the method previously described by Huang et al. [27] and Narin et al. [28], the sample (1 g) was placed in a 50 mL crucible before the addition of 10 mL of concentrated HNO_3_. The sample was heated on a hot plate until the solution became semi-dry. This was followed by the addition of 10 mL of concentrated HNO_3_. The solution was kept on a hot plate for 1 h to allow the formation of a clear suspension. After the sample was semi-dried, it was cooled and filtered through Whatman No. 42 filter paper. It was then transferred to a 50 mL volumetric flask by adding deionized distilled water to the mark [27, 29] before GF-AAS analysis.

#### Method 2 (HNO_3_ overnight digestion)

Concentrated HNO_3_ (10 mL) was added to the sample (1 g) and allowed to stand overnight at room temperature. The sample was then heated on a hot plate until the solution became clear and semi-dried. The solution was then cooled and filtered through Whatman No. 42 filter paper. It was then transferred quantitatively to a 50 mL volumetric flask by adding deionized distilled water [30]. Finally, the solution was analyzed using GF-AAS.

#### Method 3 (HNO_3_–H_2_O_2_ digestion)

In this method, the sample (1 g) was weighed into a 50 mL crucible and treated with 10 mL of concentrated HNO_3_. The solution was placed on a hot plate for 30–45 min to allow for oxidation. After cooling, 4 mL of H_2_O_2_ (20 %) was added, and the solution was reheated on a hot plate until the digest became clear and semi-dried. After cooling, the suspension was filtered into a 50 mL volumetric flask and diluted with deionized distilled water to the mark [30] before GF-AAS analysis.

#### Method 4 (HNO_3_–HClO_4_ digestion)

Approximately 1 g of sample was placed in a 50 mL crucible before the addition of 10 mL of concentrated HNO_3_. The mixture was placed on a hot plate for 30–45 min to allow for oxidation. After cooling, 5 mL of HClO_4_ (70 %) was added, and the mixture was reheated on a hot plate until the digest became clear and semi-dried. Then, the sample was cooled and filtered through Whatman No. 42 filter paper before being quantitatively transferred to a 50 mL volumetric flask by adding deionized distilled water [29, 30]. Finally, the solution was analyzed using GF-AAS.

#### Method 5 (H_2_SO_4_ digestion)

The sample (1 g) was placed in a 50 mL crucible followed by the addition of 7 mL of concentrated H_2_SO_4_. The mixture was allowed to stand for 30 min at room temperature. Approximately 7 mL of H_2_O_2_ (30 %) was added to the crucible, and the sample was reheated on the hot plate for 40 min. Thereafter, 1 mL of H_2_O_2_ (30 %) was added until the digest appeared clear upon cooling. Then, deionized distilled water was added to bring the final sample volume to 50 mL. The solution was filtered through Whatman No. 42 filter paper [29] and then analyzed using GF-AAS.

#### Method 6 (dry ashing)

Initially, 1 g of sample was placed in a crucible on a hot plate at 100–150 °C for 1 h. It was transferred to a muffle furnace set at 480 °C. After 4 h, the sample was removed from the furnace and cooled. Then, 2 mL of 5 M HNO_3_ was added, and the sample was evaporated to dryness on a hot plate. The sample was placed in a cool furnace and reheated to 400 °C for 15 min before being removed, cooled and moistened with four drops of deionized distilled water. Then, 2 mL of concentrated HCl was added, and the sample was evaporated to dryness before the addition of 2M HCl (2 mL). The solution was filtered through Whatman No. 42 filter paper and <0.45 µm Millipore filter paper and then quantitatively transferred to a 25 mL volumetric flask by adding deionized distilled water [29, 30].

### GF-AAS analysis

An atomic absorption spectrophotometer (model AA-6300, Shimadzu, Kyoto, Japan) equipped with a Shimadzu model GFA-EX7i graphite furnace atomizer was used to determine the heavy metals. Pyrolytic graphite tube was used for detection of As, Cr and Se while in case of Pb and Cd, high-density graphite tube was used. The absorption wavelength for the determination of each heavy metal type and other operating parameters and temperature programming of GF-AAS for the working elements are given in Tables 1, 2 and each analysis was performed in triplicate.

**Table 1:** Operating parameters for the GF-AAS analysis of heavy metals

Elements	Wavelength (nm)	Detection limit (µg/g)	Slit width (nm)	Lamp current (mA)	Gas flow (L/min)
Cd	228.8	0.00005	0.7	8	1
Cr	357.9	0.00002	0.7	10	1
Pb	283.3	0.00005	0.7	10	1
As	193.7	0.00010	0.7	12	1
Se	196.0	0.00005	0.7	23	1

**Table 2:** Temperature programming of GF-AAS for the analysis of Cd, Cr, Pb, As and Se in tea leaves and soil samples

Stages	Cd temperature °C, hold time (s)	Cr temperature °C, hold time (s)	Pb temperature °C, hold time (s)	As temperature °C, hold time (s)	Se temperature °C, hold time (s)
Stage-1	150, 20	150, 20	150, 20	150, 20	150, 20
Stage-2	250, 10	250, 10	250, 10	250, 10	250, 10
Stage-3	500, 10	800, 10	800, 10	600, 10	600, 10
Stage-4	500, 10	800, 10	800, 10	600, 10	600, 10
Stage-5	500, 3	800, 3	800, 3	600, 3	600, 3
Stage-6	2200, 2	2300, 2	2400, 2	2200, 2	2200, 2
Stage-7	2400, 2	2500, 2	2500, 2	2500, 2	2400, 2

### Calibration curves

Calibration curves for Cd, Cr, Pb, As and Se were prepared at seven different concentrations (0.0, 0.1, 1.0, 5.0, 10.0, 20.0 and 40.0 µg/L).

### Recovery analysis

To calculate the percent recovery, the samples were spiked with known amounts of the analytical standards of Cd, Cr, Pb, As and Se. The mean percent recoveries for the various metals were calculated using the following equation:

$${\text{ Percent recovery}} = ({\text{CE}}/{\text {CM}}) \times 100$$

where CE is the experimental concentration determined from the calibration curve, and CM is the spiked concentration.

### Determination of the transfer factor (TF)

The transfer factor or transfer coefficient was calculated by dividing the concentration of the heavy metal in present in the tea by that of the total heavy metal concentration in the soil [31]:

$${\text{ TF}} = {\text{Concentration in tea leaves}}/{\text{Concentration in soil}}.$$

## Results and discussion

### Heavy metal contents in fresh tea leaves

**Fig. 3:**
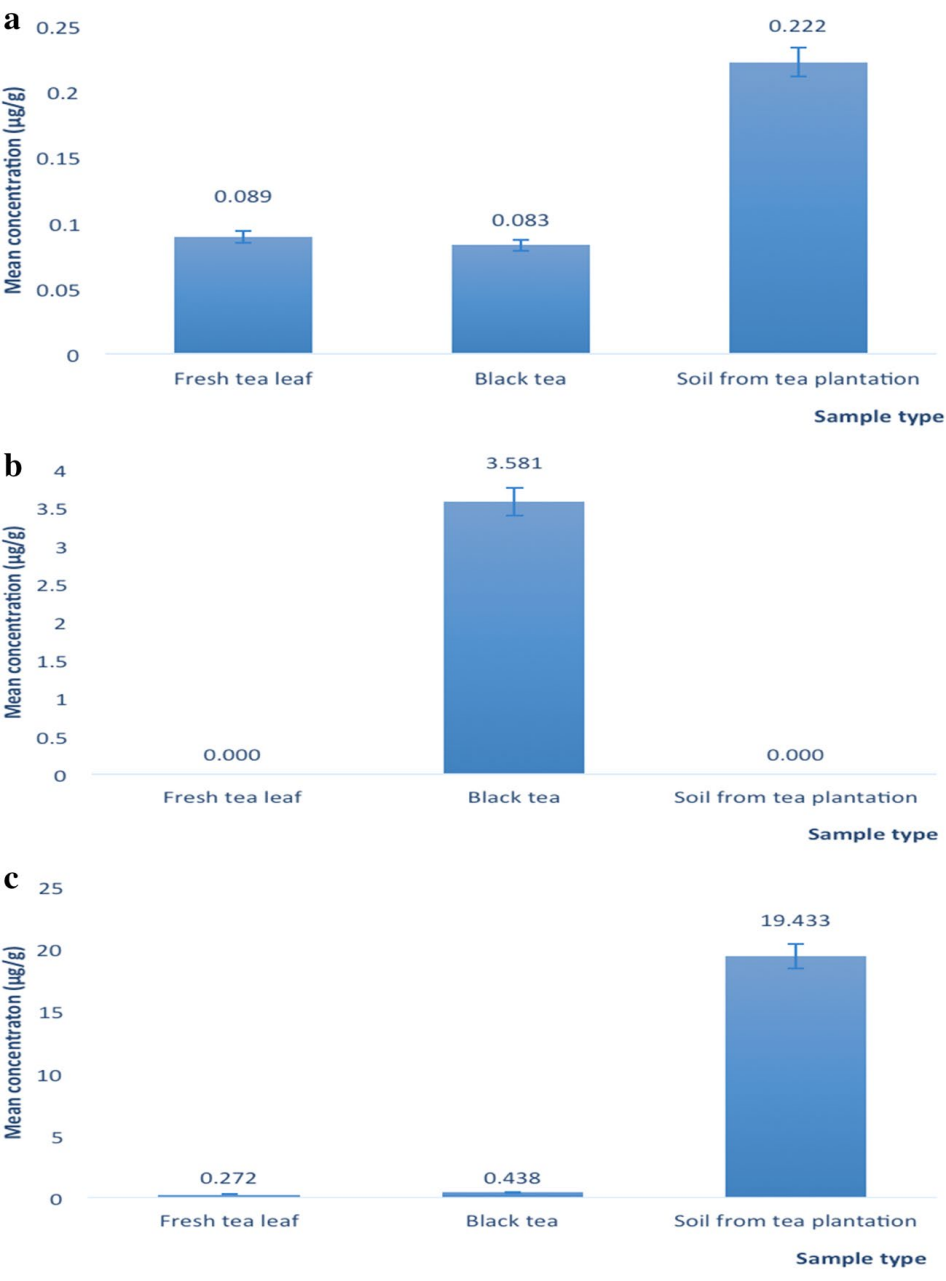
Comparison of the Cd (**a**), Cr (**b**) and Pb (**c**) content of fresh tea leaves, black tea and soil from tea plantations

Analysis of heavy metals such as As, Cr, Cd, Pb and Se in fresh tea leaves is important because they are toxic and can be transported into humans and animals via the food chain. The concentration ranges of Cd, Pb, As and Se in fresh tea leaves were (0.03–0.13), (0.05–1.14), (BDL to 2.06) and (0.47–1.31 µg/g), respectively (Table 3). Several studies have previously reported on the presence of trace elements in tea leaves and soil of tea gardens in Bangladesh [32–35]. The mean Cd concentration in fresh tea leaves was 0.09 ± 0.03 µg/g (Fig. 3), which was lower than the World Health Organization (WHO) recommended limit of 0.10 µg/g [36]. The Cd concentration was also lower than that reported for fresh tea leaves from India (0.43 ± 0.01 µg/g), China (0.77 ± 0.02 µg/g), Japan (0.15 ± 0.01 µg/g), and Italy (0.09 ± 0.01 µg/g) [37] (Table 4). Moreover, our result was also lower than Cd content of tea samples from Turkey (0.50 ± 0.10 µg/g) [28]. The variations in heavy metal contents of different samples may be due to differences in geographical location, environmental conditions, seasonal changes, physiochemical characteristics of the growing regions and matrix-to-matrix transfer.

**Table 3:** Heavy metal contents in fresh tea leaves (FTL)

Sample ID	Mean ± SD (µg/g)			
	Cd	Pb	As	Se
FTL-1	0.07 ± 0.0001	0.05 ± 0.0005	1.84 ± 0.0001	BDL
FTL-2	0.03 ± 0.0008	0.31 ± 0.0001	1.66 ± 0.0062	0.90 ± 0.0024
FTL-3	0.08 ± 0.0064	0.40 ± 0.0003	BDL	BDL
FTL-4	0.13 ± 0.0038	1.14 ± 0.0005	2.06 ± 0.0056	1.28 ± 0.0004
FTL-5	0.07 ± 0.0040	0.05 ± 0.0003	1.17 ± 0.0013	0.78 ± 0.0015
FTL-6	0.09 ± 0.0015	0.09 ± 0.0001	1.45 ± 0.0054	1.31 ± 0.0098
FTL-7	0.11 ± 0.0056	0.22 ± 0.0006	1.48 ± 0.0237	BDL
FTL-8	0.06 ± 0.0025	0.46 ± 0.0004	1.81 ± 0.0064	0.47 ± 0.0004
FTL-9	0.13 ± 0.0041	BDL	0.44 ± 0.0022	0.77 ± 0.0002
FTL-10	0.12 ± 0.0036	BDL	0.19 ± 0.0001	0.85 ± 0.0075
Mean	0.089	0.272	1.210	0.636

The limit of detection were 0.0052, 0.0026, 0.0046, 0.01 and 0.0084 µg/g for Cd, Cr, Pb, As and Se, respectively. The data (µg/g) shown in Table is reported on dry weight basis

n = 3 (*n* no. of analyses), *SD* standard deviation, *BDL* below detection limit

**Table 4:** Level of Cd, Pb, As and Se (µg/g) in tea leaves from various countries

Country	Cd	Cr	Pb	As	Se
Bangladesh	0.02 [34]	32.87 [34]	0.34 [34]	NA	ND [34]
India	0.43 [37] 0.59–0.77 [50] 0.01–0.03 [51]	0.09–0.37 [39] 1.28–1.84 [50] 0.43–1.14 [51]	1.86 [37] 0.98–1.83 [50] 0.10–0.51 [51]	NA	0.05–0.07 [39] 2.12–2.47 [51]
China	0.77 [37] 0.043 [52] 0.04–0.08 [51]	0.07–0.37 [39] 1.23–2.20 [51]	1.49 [37] 0.86 [52] 0.60–1.08 [51]	0.28 [41]	0.05–0.09 [39] 2.55–3.97 [51]
Japan	0.15 [37]	0.11–0.24 [39]	1.55 [37]	NA	0.05–0.09 [39]
Italy	0.09–0.17 [37] 0.04 [51]	1.31 [51]	0.19–0.52 [37] 0.55 [51]	NA	2.65 [51]
Turkey	0.7–0.9 [22, 28]	3.1–3.5 [22, 28]	3.1–3.7 [22, 28]	NA	
Thailand	0.001–0.086 [38]	0.040–3.294 [38]	0.108–22.245 [38]	0.013 [38] 0.010–0.238 [53]	0.00–0.01 [38] 0.014–0.508 [53]
Sri Lanka	0.03–0.24	0.05–0.11 [39]	0.59 [34]		0.05–0.09 [39] ND [34]
Iran	0.76 [50] 134.5 [54]	0.89–1.79 [50] 8.2 [54]	0.92–2.92 [50] 209.5 [54]	0.28–0.56 [41]	NA

*NA* not available data, *ND* not detected

**Fig. 4:**
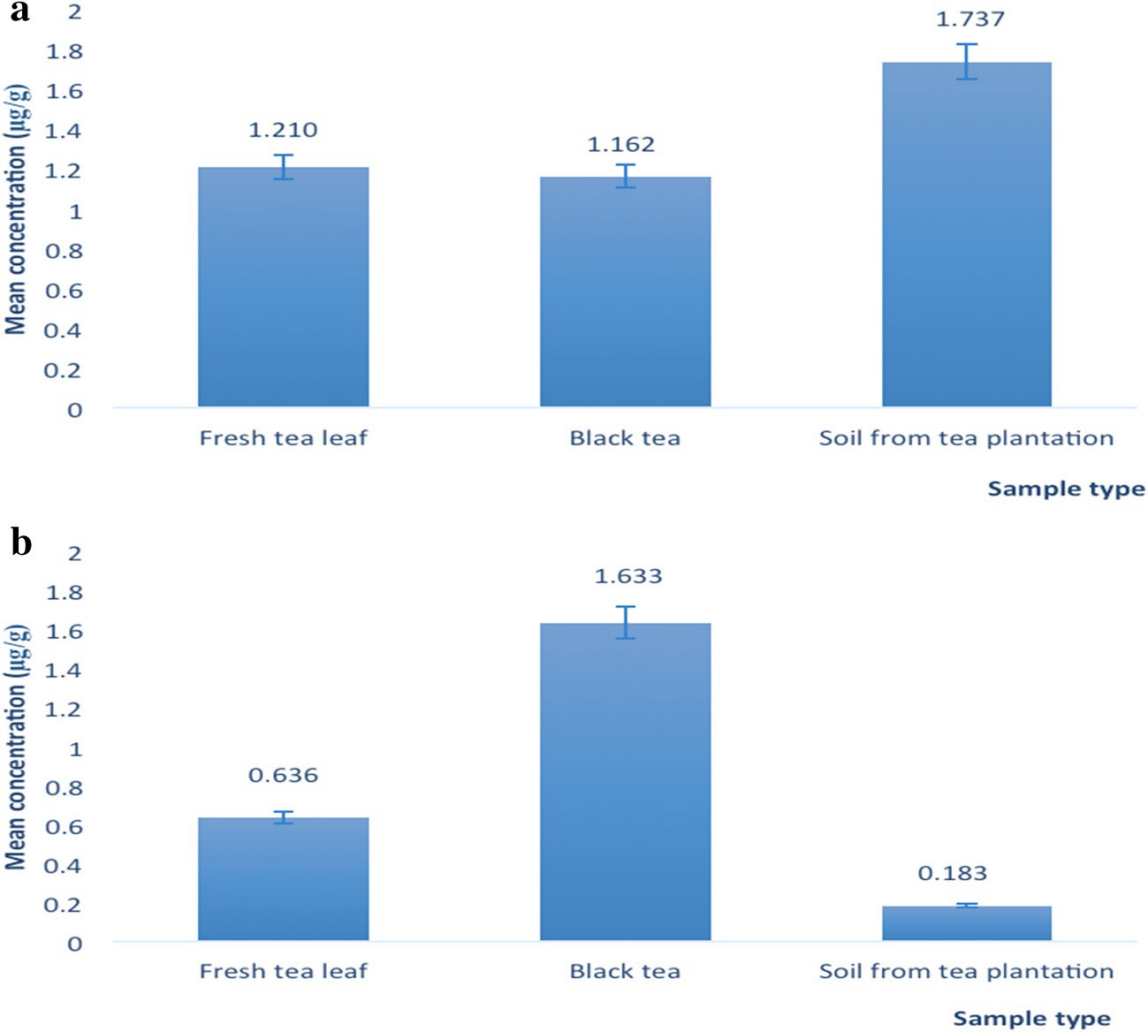
Comparison of the As (**a**) and Se (**b**) content in fresh tea leaves, black tea and soil from tea plantations

In comparison, the levels of Cr were low (below the detection limit) (Fig. 4), indicating that these fresh tea leaves were free from Cr contamination. The WHO-recommended limit for Cr is 0.05 µg/mL [36], and contamination by this heavy metal has been reported in Japanese, Chinese, Iranian and Thai green teas at 0.024, 0.14, 0.05 and 0.06 µg/g, respectively [38, 39]. Cr has been reported to cause cancer in humans, especially bronchial and lung cancers [40].

The mean Pb concentration in all of the fresh tea leaves investigated was 0.27 ± 0.35 µg/g (Fig. 3), which is lower than the WHO-recommended limit of 0.30 µg/g [36]. This is also lower than the Pb content of tea leaves from Turkey (17.90 ± 7.10 µg/g) [22] as well as tea leaves from India (1.86 ± 0.04 µg/g), China (1.49 ± 0.03 µg/g) and Japan (1.55 ± 0.03 µg/g), but is slightly higher than that from Italy (0.23 ± 0.01 µg/g) [37]. Pb is a cumulative toxin that can primarily affect the blood, nervous system and kidneys. If present in high concentrations, Pb inhibits red blood cell formation, which can result in anemia [36].

The mean As concentration in fresh tea leaves was 1.21 ± 0.74 µg/g (Fig. 4), which is higher than the WHO-recommended limit (0.10 µg/g) [36] and higher than that of green tea from China (0.28 µg/g) [41], Thailand (0.013 µg/g) [38], Canada (0.04 µg/g) [42] and Japan (0.00 µg/g). A potential source of As is the high amount of As present in the soils of the studied tea plantations. As is toxic to humans, especially in its methylated forms produced by glutathione s-transferase (GST), As III methyltransferase (AS3MT) and S-adenosyl methionine (SAM). These enzymes can compete with DNA methyltransferase (DNMT) for DNA methylation, hence indirectly inhibiting DNA methyltransferase and inducing the reactivation of silenced tumor suppressor genes (Mishra et al. 2009). Chronic toxicity from high exposure to inorganic As is associated with arsenicosis, melanosis, keratoses of the skin and cancer [36].

The Se content of all investigated fresh tea leaves was 0.64 ± 0.50 µg/g (Fig. 4), and the WHO-recommended limit and contents of Japanese sencha green tea, Japanese jasmine tea, Chinese pai mu tan tea and Chinese gunpowder tea were 0.125, 0.092, 0.089, 0.075 and 0.070 µg/g, respectively [39]. Se can lead to selenosis if taken in doses exceeding 400 µg per day [43]. Symptoms of selenosis include a garlic odor of the breath, gastrointestinal disorders, hair loss, sloughing of nails, fatigue, irritability and neurological damage. Extreme cases of selenosis can result in cirrhosis of the liver, pulmonary edema and death [43].

### Heavy metal contents in black tea

In the present study, heavy metal contents were also analyzed in the black tea produced from Bangladesh. The concentration ranges of Cd, Cr, Pb, As and Se were 0.04–0.16, 0.45–10.73, 0.07–1.03, 0.89–1.90 and 0.76–10.79 µg/g, respectively using HNO_3_ overnight digestion procedure (Table 5).

**Table 5:** Heavy metal contents in processed tea leaves (PTL, black tea)

Sample ID	Mean ± SD (µg/g)				
	Cd	Cr	Pb	As	Se
PTL-1	0.16 ± 0.0013	9.31 ± 0.0493	0.27 ± 0.0008	1.90 ± 0.0006	1.44 ± 0.0038
PTL-2	0.10 ± 0.0031	7.03 ± 0.0156	0.70 ± 0.0004	BDL	BDL
PTL-3	0.12 ± 0.0023	10.73 ± 0.0348	0.40 ± 0.0009	1.17 ± 0.0153	0.80 ± 0.0002
PTL-4	0.04 ± 0.0030	2.10 ± 0.0004	0.07 ± 0.0021	1.40 ± 0.0036	0.76 ± 0.0023
PTL-5	0.06 ± 0.0031	1.71 ± 0.0032	0.31 ± 0.0008	0.89 ± 0.0017	BDL
PTL-6	0.11 ± 0.0030	0.45 ± 0.0026	0.22 ± 0.0006	1.78 ± 0.0066	10.79 ± 0.0065
PTL-7	0.05 ± 0.0001	2.75 ± 0.0086	0.66 ± 0.0002	1.02 ± 0.0030	0.44 ± 0.0003
PTL-8	0.07 ± 0.0004	1.19 ± 0.0084	1.03 ± 0.0011	1.16 ± 0.0006	BDL
PTL-9	0.05 ± 0.0035	BDL	0.72 ± 0.0006	1.00 ± 0.0060	1.89 ± 0.0101
PTL-10	0.07 ± 0.0020	0.54 ± 0.0049	BDL	1.30 ± 0.0004	0.21 ± 0.0016
Mean	0.083	3.581	0.438	1.162	1.633

The limit of detection were 0.0045, 0.003, 0.0028, 0.0032 and 0.0064 µg/g for Cd, Cr, Pb, As and Se, respectively. The data (µg/g) shown in Table is reported on dry weight basis

n = 3 (*n* no. of analyses), *SD* standard deviation, *BDL* below detection limit

The mean concentration of Cd in black tea (0.08 ± 0.04 µg/g) (Fig. 3) was lower than the World Health Organization (WHO)-recommended limit of 0.10 µg/g [36], but higher than that reported in black tea from Canada (0.026 µg/g) [42], Thailand (0.0071 µg/g) [41] and Turkey (0.0100 µg/g) [44]. However, its level was lower than that reported in India (0.8900 µg/g) [3], Nigeria (0.1200 µg/g) and Saudi Arabia (0.9890 µg/g) [41]. Moreover, our result was also lower than Cd content of black teas from Turkey (2.30 ± 0.40 µg/g) [22]. In a previous study, the concentration of Cd was 0.03 µg/g [34] which is slightly lower than that of our findings. In another study, the presence of some trace elements (Cu, Ni, Mn and Zn) in three commercially available tea from Bangladesh were analyzed [32]. Nevertheless, they were different from that of the current investigation.

Cr was detected in rather high amounts in black tea (3.581 ± 3.941 µg/g), but it was not detected in fresh tea leaves or tea plantation soils (Fig. 3). Its level is higher than the recommended limit for Cr by the WHO of 0.05 µg/mL [36]. Moreover, Cr concentrations in black tea from India, China, Sri Lanka and Turkey were reported at 0.371, 0.155, 0.050 and 3.000 µg/g [39, 44], respectively. It is plausible that Cr contamination occurred during the fermentation process, which is one of the important processing steps of black tea in Bangladesh. In particular, it may occur during the CTC rolling steps involved in the production of black tea. However, this finding is lower than the previously reported Cr concentration (32.87 µg/g) in some tea samples from Bangladesh [34] which may be contributed to the different types of tea samples used as well as variance in the type of soil in the tea garden.

The Pb concentration in black tea was 0.438 ± 0.328 µg/g (Fig. 3), which is higher than the WHO recommended limit of 0.30 µg/g [36]. Moreover, our findings are also similar to the previously reported concentration of Pb (0.34 µg/g) [34] in tea samples from Bangladesh but is higher than those reported for Nigeria (0.330 µg/g) [6], Egypt (0.395 µg/g) and Thailand (0.0237 µg/g) [41], but lower than that in Turkey (2.500 µg/g) [44], Iran (2.915 µg/g), Saudi Arabia (1.250 µg/g), China (3.270 µg/g), Pakistan (2.500 µg/g) and India (0.810 µg/g) [41].

The mean As concentration in black tea was 1.162 ± 0.524 µg/g (Fig. 4, which was higher than the WHO-recommended limit (0.10 µg/g) [36], as well as higher than in Thailand (0.00084 µg/g) and China (0.280 µg/g) [41]. However, it was lower than that reported in Nigeria (2.220 µg/g) [6]. The Se content in black tea from Bangladesh was higher (1.633 ± 3.280 µg/g) (Fig. 4) than that reported in black tea from Nigeria [6], India, China and Sri Lanka [39], which were 0.520, 0.070, 0.087 and 0.050 µg/g, respectively.

### Heavy metal contents in soils from tea plantations

In this part of the study, the heavy metal contents in the soils from tea plantations in Bangladesh have been reported. This analysis is important because of the metals’ potential toxicity and transportation through the root system into the buds and tea leaves. The concentration ranges of Cd, Pb, As and Se in tea plantation soils were 0.11–0.45, 2.80–66.54, 0.78–4.49 and 0.03–0.99 µg/g, respectively (Table 6).

**Table 6:** Heavy metal contents in soils from tea plantations (STP)

Sample ID	Mean ± SD (µg/g)				
	Cd	Cr	Pb	As	Se
STP-1	0.16 ± 0.0013	BDL	66.54 ± 0.5200	BDL	BDL
STP-2	0.15 ± 0.0042	BDL	10.65 ± 0.0120	2.75 ± 0.0013	BDL
STP-3	0.34 ± 0.0019	BDL	63.63 ± 4.2400	1.13 ± 0.0041	BDL
STP-4	0.24 ± 0.0016	BDL	8.65 ± 0.0420	4.49 ± 0.0128	BDL
STP-5	0.22 ± 0.0021	BDL	11.86 ± 0.0660	1.79 ± 0.0017	BDL
STP-6	0.45 ± 0.0015	BDL	6.90 ± 0.2000	2.32 ± 0.0022	BDL
STP-7	0.16 ± 0.0038	BDL	2.80 ± 0.0100	1.08 ± 0.0117	0.03 ± 0.0003
STP-8	0.23 ± 0.0015	BDL	9.48 ± 0.0260	3.03 ± 0.0004	BDL
STP-9	0.16 ± 0.0011	BDL	3.60 ± 0.0350	BDL	0.99 ± 0.0000
STP-10	0.11 ± 0.0059	BDL	10.22 ± 0.4400	0.78 ± 0.0157	0.81 ± 0.0032
Mean	0.222	–	19.433	1.737	0.183

The limit of detection were 0.036, 0.0018, 0.0093, 0.0051 and 0.0012 µg/g for Cd, Cr, Pb, As and Se, respectively. The data (µg/g) shown in Table is reported on dry weight basis

n = 3 (*n* no. of analyses), *SD* standard deviation, *BDL* below detection limit

Similar to the findings for fresh tea leaves, Cr was not detected in the tea garden soil samples (Fig. 4). However, Cr has been reported in agricultural soils in the United States (48.5 µg/g) [45], India (1.23 µg/g) [46] and Kunshan, China (87.73 µg/g) [47]. Low concentrations of Cd (mean 0.222 ± 0.103 µg/g) were observed in all investigated soils from the tea plantations samples (Fig. 3). These levels were lower than that previously reported in U.S. agricultural soils (13.5 µg/g) [45], but higher than in Indian agricultural soils (0.05 µg/g) [46] and soil from Kunshan in China (0.20 µg/g) [47].

Because of the toxicological importance of Pb, many studies have investigated the levels of this element in soil from several countries. Among all of the soil samples investigated, STP-1 had the highest Pb concentration (66.54 ± 0.520 µg/g) potentially because of its location, which was adjacent to a highway. Overall, the mean level of Pb in the tea plantation soil samples was 19.43 ± 24.25 µg/g (Fig. 3). This is higher than that reported for agricultural soils in India (2.82 µg/g) [46] but lower than agricultural soils in the U.S. (55.00 µg/g) [45] and Kunshan, China (30.48 µg/g) [47].

The concentrations of As ranged from 0.78 to 4.49 µg/g. The highest As level was 4.49 µg/g in STG-4, but As was not detected in STP-1 or STP-9. The mean concentration of As was 1.74 ± 1.429 µg/g (Fig. 4), which is lower than that reported in Kunshan, China (8.15 µg/g) [47]. Among all of the investigated soil samples, the mean Se concentrations in STP-1, STP-2, STP-3, STP-4, STP-5, STP-6 and STP-8 were below the detection limit. Low Se contents (mean 0.18 ± 0.398 µg/g) (Fig. 4) have also been reported in soils from garlic (0.026 µg/g), radish (0.028 µg/g), carrot (0.011 µg/g) and orchard grass (0.069 µg/g) plantations [48]. In comparison, higher Se concentrations were detected in the soils of oilseed rape (0.316 µg/g), white clover (0.211 µg/g), red clover (0.223 µg/g) and English plantain (0.277 µg/g) plantations [48]. These higher Se concentrations may be attributed to fertilizer (sodium selenite) use in tea plantations. High levels of heavy metals such as Se and As can potentially be easily transported to the tea leaves through the roots of the plant from contaminated soils. In addition, the acidic nature of tea garden soils can increase the extraction of As and hence the detected As concentration.

### Heavy metal transfer from soils to tea leaves in Bangladesh

Soil-to-plant transfer is one of the key components of human exposure to metals through the food chain. The transfer factor (TF) describes the transfer of heavy metals from soils to the plant body. In the present study, the TFs for Cd, Pb, As and Se were 0.47845, 0.03122, 0.45524 and 0.18272, respectively (Table 7). The transfer factors for heavy metals in the investigated tea samples decreased as follows: Cd > As > Se > Pb. In general, the TFs increased with decreasing metal concentrations in soils. Thereby, lower TFs in tea plants could be explained by uptake saturation [49]. In another study, the TFs of lettuce, spinach, radish and carrot followed a trend of Mn > Zn > Cd > Pb (Intawongse and Dean, 2006). To our knowledge, our study is the first to report TFs in tea.

**Table 7:** Transfer factors of heavy metals from tea plantation soils of tea leaves

Sample ID	Cd	Pb	As	Se
FTL-1	0.4375	0.0008	–	–
FTL-2	0.2000	0.0291	0.6036	–
FTL-3	0.2353	0.0062	–	–
FTL-4	0.5417	0.1318	0.4588	–
FTL-5	0.3182	0.0042	0.6536	–
FTL-6	0.2000	0.0130	0.6250	–
FTL-7	0.6875	0.0786	1.3704	–
FTL-8	0.2609	0.0485	0.5974	–
FTL-9	0.8125	0.0000	0.0000	0.7778
FTL-10	1.0909	0.0000	0.2436	1.0494
Mean	0.4784	0.0312	0.4552	0.1827

### Method validation

The analytical results for the recovery of spiked metals in tea using the six digestion methods and LODs for each method are presented in Table 8. Method 2 (overnight digestion with HNO_3_) was the most efficient for recovering Cd, Cr, Pb, As and Se with mean percent recoveries of 99.50, 97.30, 100.00, 89.30 and 100.03 %, respectively. For this reason, all tea samples were subsequently digested using this method, which is recommended as the best method for the destruction of tea. The method likely provided sufficient time for HNO_3_ to digest the tea matrix. On the other hand, Method 5 (H_2_SO_4_) yielded the lowest recoveries, possibly due to the incomplete digestion of tea samples or losses of elements through volatilization. Recoveries of Cd, Cr, Pb, As and Se were 80.60, 56.00, 65.00, 58.80 and 71.20 %, respectively, all of which were below the acceptable limits (75–125 %), except for Cd (80.60 %). Thus, the digestion method using H_2_SO_4_ is not recommended for tea samples. However, in a previous study, tea samples digested with three different acids at similar ratio [HNO_3_/H_2_SO_4_/H_2_O_2_(2: 2: 2)] showed shorter digestion time with better recovery and precision than other acid mixtures [28].

**Table 8:** Recovery analysis (n = 2) of heavy metals and LODs of investigated methods for method validation

Method	Percentage, LOD (µg/g)				
	Cd	Cr	Pb	As	Se
Method 1 (HNO_3_)	96.80, 0.0076	95.20, 0.094	70.70, 0.012	80.00, 0.152	72.50, 0.0049
Method 2 (HNO_3_ overnight)	99.50, 0.0052	97.30, 0.0026	100.00, 0.0047	89.30, 0.014	100.03, 0.0084
Method 3 (HNO_3_ and H_2_O_2_)	94.80, 0.016	74.90, 0.062	75.80, 0.186	95.60, 0.021	72.00, 0.0124
Method 4 (HNO_3_ –HClO_4_)	76.20, 0.002	93.50, 0.068	74.90, 0.0052	90.20, 0.065	86.10, 0.0325
Method 5 (H_2_SO_4_)	80.60, 0.018	56.00, 0.128	65.00, 0.194	58.80, 0.176	71.20, 0.0982
Method 6 (Dry ashing)	113.60, 0.0142	87.40, 0.0014	84.70, 0.024	93.20, 0.052	60.20, 0.0018

The uncertainty of results was less than 1 %. The data (µg/g) shown in Table is reported on dry weight basis

Method 1 (destruction with HNO_3_) and Method 3 (digestion using HNO_3_ and H_2_O_2_) yielded acceptable recoveries of Cd, Cr, Pb, As and Se. However, only 70.70 % of Pb was recovered by Method 1, which is below the acceptable limit. Therefore, Methods 1 and 3 could also be used as relatively inexpensive, simple and rapid substitutes. Method 6 (Dry ashing) is not recommended because of the high cost incurred due to the requirement of a muffle furnace. Method 4 (HNO_3_–HClO_4_ procedure) is also not recommended because HClO_4_ is potentially hazardous during digestion. This method also yielded poor recoveries. For all procedures, recovery of Cd was significantly higher, while recovery of Pb was relatively lower. The likely reason for the lower recovery of Pb is the effect of the acidic pH used during sample digestion, which does not favor sample extraction.

## Conclusions

Six digestion methods followed by GF-AAS have been successfully optimized in the present study. An overnight digestion with nitric acid (method no. 2) offered adequate time to digest the tea matrix and was the most efficient method for recovering Cd, Cr, Pb, As and Se. Moreover, Methods 1 and 3 were also satisfactory, relatively cheap, simple and fast. Method no. 5 is not recommended for the digestion of tea samples while method no. 6 was expensive. Cd, Pb, As and Se were detected in fresh tea leaves, but Cr was not detected. The concentrations of As were high in both fresh and black tea, while the concentration of Pb and Cr in black tea was higher than the recommended level set by the WHO. The soil from tea plantations was contaminated with As and Se, levels of which were at times higher than the WHO recommendation. High levels of heavy metals can easily be transported to tea leaves through the roots of tea plants. However, Cr was not detected in the soil samples. The trend in heavy metal TFs in the investigated tea samples was Cd > As > Se > Pb. An overnight digestion with HNO_3_ was the most efficient digestion method for recovering heavy metals.
